# Tomato (*Solanum lycopersicum* L.) as a Source of Bioactive Compounds: Functional Properties and Technological Aspects—A Review

**DOI:** 10.3390/nu18132084

**Published:** 2026-06-25

**Authors:** Anna Bajon, Marcin Kidoń, Joanna Kobus-Cisowska

**Affiliations:** 1 Department of Food Quality and Safety Management, Faculty of Food Technology and Human Nutrition, Poznań University of Life Sciences, Wojska Polskiego Street 31/33, 60-624 Poznań, Poland; anna.bajon@up.poznan.pl; 2 Department of Food Technology of Plant Origin, Faculty of Food Technology and Human Nutrition, Poznań University of Life Sciences, Wojska Polskiego Street 31/33, 60-624 Poznań, Poland; marcin.kidon@up.poznan.pl; 3 Department of Gastronomy Science and Functional Foods, Faculty of Food Technology and Human Nutrition, Poznań University of Life Sciences, Wojska Polskiego Street 31/33, 60-624 Poznań, Poland

**Keywords:** tomato, carotenoids, polyphenols, nutritional value, source of variation, health impact

## Abstract

Tomato (*Solanum lycopersicum* L.), a member of the Solanaceae family, originates from South America and is currently cultivated worldwide. In tropical regions, it grows as a perennial plant, whereas in temperate and subtropical climates, it is cultivated as an annual because of its sensitivity to frost. Tomato fruits are an important source of bioactive compounds, including carotenoids, polyphenols, minerals, and vitamins. Their widespread consumption and status as one of the most commonly consumed horticultural crops worldwide make tomatoes an important dietary source of these compounds. Tomatoes are commonly consumed fresh, but they are also an important raw material for the food industry. The main tomato products include juices, concentrates, purées, and sauces. The chemical composition and concentration of bioactive compounds in tomato fruits depend on several factors, including cultivar, stage of ripeness, environmental conditions, and cultivation and processing technologies. Numerous studies indicate that compounds present in tomatoes exhibit antioxidant properties and have been associated with potential health-promoting effects. Among these, carotenoids, particularly lycopene, play a key role. This review summarizes current knowledge on the nutritional value, composition, and functional properties of tomatoes. It also addresses the antinutritional aspects of tomato compounds, as well as the influence of agrotechnical, environmental, and technological factors on the content of bioactive compounds. Furthermore, this review may support the design of future research by critically analyzing existing studies and highlighting inconsistencies and knowledge gaps.

## 1. Introduction

The tomato (*Solanum lycopersicum* L.) is an edible plant belonging to the Solanaceae family, commonly known as the nightshade family. This species originates from South America; however, tomatoes are now cultivated worldwide. In intertropical climates, tomatoes behave as perennial plants, whereas in temperate and subtropical regions, they are cultivated as annuals because of their sensitivity to frost. The growing season may be extended by cultivation under foil tunnels or greenhouses. Tomatoes are commonly consumed raw but are also a highly valued raw material in the food industry due to their nutritional value, attractive sensory properties, and versatility in processing. The most common tomato products include juices and juice concentrates, purées, sauces, and whole, cut, or mashed fruits pasteurized in cans [1].

Although tomato is commonly regarded as a vegetable, its edible part is botanically classified as a fruit, specifically a berry. Tomato fruits are an important source of vitamins and minerals and play a significant role in a balanced human diet. Numerous studies have confirmed that tomatoes are rich in macronutrients, micronutrients, and bioactive compounds, among which carotenoids and polyphenolic compounds are particularly important. Due to this composition, tomatoes not only provide essential nutrients but also support many physiological processes in the human body [2,3].

The aim of this review was to evaluate tomato fruit as a raw material for the food industry with high nutritional and functional value, with particular emphasis on its chemical composition and content of bioactive compounds. The review focuses on agrotechnical and environmental factors affecting fruit quality, mineral composition, and the presence of bioactive compounds, particularly carotenoids, polyphenols, vitamins, and potentially toxic compounds. Additional attention was given to the influence of cultivar, cultivation conditions, and technological processing on the content and bioavailability of these compounds in fresh and processed tomato products, as well as to their antioxidant and potential health-promoting properties. The collected literature data highlight the importance of tomatoes in human nutrition and their functional potential in this context. Furthermore, this review may support the design of future research by critically analyzing existing studies and highlighting inconsistencies and knowledge gaps.

## 2. Literature Search Strategy and Data Presentation

All data presented in this review were compiled from scientific literature. These sources were systematically searched using electronic databases, including ScienceDirect, PubMed, CNKI, Web of Science, Scopus, Google Scholar, and SciELO. The literature search was conducted using the keywords “tomato”, “*Solanum lycopersicum*”, “carotenoids”, “phenolic compounds”, “bioactive compounds”, “ascorbic acid”, “tocopherol”, and “toxicity”. These search terms were used individually and in combination.

Studies were included if they were original peer-reviewed research articles published in English between 2000 and 2026 and provided qualitative or quantitative data on the chemical composition, bioactive compounds, nutritional value, cultivation practices, cultivar diversity, processing, biological activity, or safety aspects of tomato fruits and tomato-derived products. Both *in vitro* and *in vivo* studies were considered.

Conference abstracts, editorials, book chapters, duplicate records, and non-English publications were excluded from further analysis.

To facilitate data comparison among studies, values originally reported on a dry matter basis were converted to a fresh matter (FM) basis. When dry matter content was not specified in the original publication, a moisture content of 94% was assumed for the conversion.

## 3. Proximate Composition of Tomato Fruits

Tomatoes are popular vegetables consumed for their taste and high nutritional value. The composition of tomato fruit varies depending on several factors, mainly cultivar, growing conditions, stage of maturity, and fertilization practices. The main component of tomato fruit is water, which constitutes approximately 89–95% of its total weight (Table 1). The content of soluble solids also varies considerably, ranging from 3.0 to 9.2 °Brix.

An effect of fruit size on total soluble solids (TSS) can be observed. As reported in [4], the small-sized tomato cultivar ‘Cherry’ (average fruit mass 3.24 ± 0.48 g) contained 0.7–3.7 °Brix more soluble solids than the large-fruited cultivars ‘Monika’ and ‘Isabella’ (average fruit mass 43.68 ± 8.92 g and 47.23 ± 10.2 g, respectively). Similarly, Ref. [5] showed that cherry cultivars had higher soluble solids than regular cultivars.

In general, TSS increases during tomato maturation and ripening. For example, during maturation from the green stage to the deep red stage, the soluble solids content may increase by around 45% [4]. This increase is typical for tomatoes because during ripening, starch and other polysaccharides that accumulated during early fruit development are converted into reducing sugars, mainly fructose and glucose [6]. In addition, sucrose transported from leaves to the fruit is hydrolyzed into glucose and fructose, which directly contributes to higher refractometric readings. The content of simple sugars is also positively correlated with the perception of sweetness [7,8]. Environmental stress may further increase the soluble solids and sugar content. For example, Ref. [9] reported that tomatoes cultivated under water-deficit conditions showed higher TSS and sugar accumulation than control fruits.

The typical acidity of tomato fruits ranges from 0.17 to 0.89 g/100 g fresh mass (FM). The main contributors to tomato acidity are citric and malic acids, although oxalic acid has also been reported by some authors [7]. The pH of tomato fruits generally ranges from 3.5 to 4.6 depending on cultivar and maturity stage; however, acidity usually does not change markedly during ripening.

**Table 1:** Basic physicochemical composition of different tomato cultivars growth under different conditions reported in the literature.

Varieties	Additional Information	Water Content %	TSS °Brix	pH	Acidity g/100 g	Reference
Seven regular varieties	Greenhouse, Republic of Korea	NA	4.10–5.13	4.27–4.58	0.17–0.25	[5]
Thirteen cherry varieties	Greenhouse, Republic of Korea	NA	6.07–8.77	4.30–4.56	0.21–0.27	[5]
Six fresh market varieties	Open-field, harvested at mature green stage, Ethiopia	NA	4.2–5.2	4.08–4.44	0.788–0.889	[10]
Three processing varieties Roma VF, Melkasalsa, Melkashola	Open-field, harvested at mature green stage, Ethiopia	NA	4.1–4.2	3.37–3.96	0.748–0.756	[10]
Four varieties: Argeş 11, Argeş 123, Costate 21, Stefănești 22	Plastic tunnel, organic conditions, Romania	93.22–95.14	3.4–4.8	3.87–4.16	0.473–0.587	[11]
Small-size variety Cherry	Greenhouse, range during fruit ripening from mature green to deep red, Oman	88.8–89.7	6.0–8.8	3.5–3.8	NA	[4]
Large-size variety Monika	Greenhouse, range during fruit ripening from mature green to deep red, Oman	92.6–93.7	5.0–5.6	4.0–4.3	NA	[4]
Large-size variety Isabella	Greenhouse, range during fruit ripening from mature green to deep red, Oman,	91.8–93.2	5.3–7.6	3.9–4.3	NA	[4]
Four varieties: Bishola, Eshete, Marglobe, Money maker	Open-field, Ethiopia	NA	4.10–4.56	4.09	0.55–0.78	[12]
	Greenhouse, Ethiopia	NA	3.00–4.20	4.10	0.48–0.58	[12]
Three genotypes: Carmen, Santa Clara, BGH-320 accession	Open-field, Brazil	NA	5.20–5.93	4.37–4.59	0.30–0.44	[13]
	Non-heated greenhouse, Brazil	NA	3.60–3.83	4.34–4.56	0.26–0.37	[13]
Ten varieties	Open-field, organic cultivation, Spain	93.39–95.93	4.3–9.2	NA	0.34–0.67	[14]
Eight varieties: seven traditional and one commercial	Open-field, organic cultivation, harvested at red ripening stage, Spain	NA	4.05–6.22	4.15–4.34	0.24–0.35	[15]

NA—not available.

Tomatoes are low-calorie foods, providing approximately 15–25 kcal per 100 g FM, and they have a low glycemic index (GI around 15) [16]. They are characterized by high water content and relatively low levels of macronutrients. Fat content is generally very low, typically ranging from 0.10 to 0.94 g/100 g FM (Table 2). Only one paper presented higher (to 2.89 g/100 g FM) values. Protein levels reported in the literature usually range from 0.10 to 1.00 g/100 g FM, although some studies have reported higher values of 1.32–2.55 g/100 g [4,17]. Higher protein levels have also been observed in small-fruited cultivars compared with large-fruited ones [4].

Tomato fruits also contain considerable amounts of free amino acids, particularly glutamic acid (Glu), aspartic acid (Asp), γ-aminobutyric acid (GABA), and glutamine (Gln), which together may account for 50–70% of the total free amino acids. Glutamic and aspartic acids contribute to the characteristic umami and sour-umami taste of tomatoes and are strongly associated with their flavor profile [18,19,20].

Carbohydrate content in fresh tomatoes typically ranges from 1.01 to 7.23 g/100 g FM. The predominant sugars in ripe fruits are glucose and fructose, whereas small amounts of sucrose may also occur, particularly in cherry-type cultivars [14]. Dietary fiber content ranges from 0.14 to 1.69 g/100 g FM depending on cultivar and geographical origin. According to the USDA FoodData Central database, raw red tomatoes contain approximately 0.88 g protein, 0.20 g fat, 3.89 g carbohydrates, and 1.2 g dietary fiber per 100 g of FM. These values fall within the ranges reported in the literature [21].

**Table 2:** Proximate composition of tomato fruits reported in the literature (energy kcal/100 g FM, others g/100 g FM).

Varieties	Additional Information	Energy	Protein	Fat	Carbohydrates	Dietary Fiber	Reference
Seven varieties	Open-field Nigeria	8.9–13.42 ^2^	1.56–2.55	0.21–0.25	NA	0.36–0.45	[17]
Two varieties: Astra, Eureka	Greenhouse experiment, elevated vs. ambient CO_2_, mature fruits	14.0–18.62 ^2^	0.63–0.921	0.11–0.201	2.31–2.931	0.60–0.681	[22] ^1^
Small-size variety Cherry	Greenhouse, range during fruit ripening from mature green to deep red	NA	1.32–1.46	1.28–2.89	NA	1.30–1.55	[4]
Large-size variety Monika	Greenhouse, range during fruit ripening from mature green to deep red	NA	0.44–0.87	0.13–0.74	NA	0.61–0.92	[4]
Large-size variety Isabella	Greenhouse, range during fruit ripening from mature green to deep red	NA	0.68–0.94	0.31–0.51	NA	0.68–0.90	[4]
Four varieties	Fruit possessed from Nigerian market	17.1–30.32 ^2^	0.91–1.00	0.91–0.94	2.72–3.62	1.24–1.69	[23,24]
Ten varieties	Open-field, organic cultivation, Spain	NA	0.28–0.57	NA	2.81–4.59	NA	[14]
Hausa Yoruba	Local Nigerian landraces, stored fruits (20 days)	22.5–24.82 ^2^	0.10–0.11	0.10–0.13	5.23–5.72	0.14–0.15	[19]
Tropimech Roma VF	Improved varieties, stored fruits (20 days)	16.4–34.72 ^2^	0.15–0.55	0.10–0.14	3.65–7.23	0.16–1.14	[19]
Eight varieties	Greenhouse, Spain, different ripening stage from pink to light red	8.9–16.2 ^2^	0.55–1.05	0.20–0.67	1.01–2.18	0.74–1.60	[25]
Tomato red, ripe	Raw, edible portion, average tomato	18	0.88	0.20	3.89	1.20	[21]
Tomato Roma/plum type	Raw Roma plum-type tomato	21	0.90	0.20	4.20	1.20	[21]
Tomato cherry type	Raw cherry tomato, small-fruited variety	18	0.88	0.20	3.90	1.20	[21]
Tomato yellow	Raw yellow tomato variety	15	0.90	0.20	3.30	1.10	[21]
Tomato orange	Raw orange tomato variety	25	1.00	0.30	5.00	1.50	[21]
Tomato green, unripe	Raw green tomato	15	1.00	0.20	3.50	1.10	[21]

^1^ Values reported on a dry matter (DM) basis in the original studies were converted to a fresh matter (FM) basis assuming 94% moisture content. ^2^ Values calculated from proxime composition. NA—not available.

## 4. Mineral Composition

Tomatoes are a relevant source of both macro- and microelements in the human diet. As shown in Table 3, potassium (K) is the dominant mineral in tomato fruits. Its content typically ranges from 126 to nearly 284 mg/100 g FM, although a few studies have reported values below 80 mg/100 g FM. According to the European Food Safety Authority (EFSA), the adequate intake (AI) for potassium in adults is 3500 mg/day [26]. Thus, the consumption of 100 g of tomatoes provides approximately 4–8% of this recommendation. Although this contribution is moderate, tomatoes are widely consumed both fresh and in processed forms, which increases their dietary importance.

Other macrominerals present in tomatoes include phosphorus (P), magnesium (Mg), and calcium (Ca), but in considerably lower concentrations. Sodium (Na) levels are generally very low, usually below 10 mg/100 g FM. As a result, a 100 g portion of tomatoes provides less than 0.5% of the recommended daily sodium intake. Only the study reported in [27] found higher sodium concentrations (36–63 mg/100 g FM).

Among the trace elements, iron (Fe), zinc (Zn), copper (Cu), and manganese (Mn) are noteworthy, although the Fe and Mn concentrations reported by [22] appear considerably higher than typical literature values. Ref. [28] evaluated the *in vitro* bioaccessibility of minerals from tomatoes. Among the macrominerals, magnesium was more bioaccessible than calcium and potassium. Among the trace elements, copper showed the highest bioaccessibility. Although tomatoes are not a major source of these minerals, their frequent consumption, particularly in Mediterranean diets, may contribute to daily mineral intake.

Variability in mineral composition is mainly associated with genotype, fertilization regime, cultivation system, and environmental conditions. Slightly higher mineral contents are often reported in greenhouse systems than in open-field cultivation, possibly due to more intensive fertilization and higher mineral concentrations in potting substrates. Soil salinity may also influence mineral composition. For example, Ref. [29] reported that increasing irrigation water salinity increased the sodium and chlorine levels but reduced the phosphorus, potassium, magnesium, copper, and zinc concentrations in tomato fruits, likely because of ion competition under saline conditions. However, high salinity is generally not recommended because it negatively affects plant growth and crop yield [30].

Consumption of tomatoes is associated with a relatively low risk of heavy metal intake, including lead (Pb), chromium (Cr), cadmium (Cd), and nickel (Ni). Although plants grown in contaminated environments may accumulate higher levels of these elements, they are mainly retained in the roots, leaves, and stems, while their accumulation in fruits is usually much lower and not raise any health concerns [31,32].

**Table 3:** Mineral composition (mg/100 g FM) of tomato fruits reported in the literature.

Sample Type	N	P	K	Mg	Ca	Na	Fe	Zn	Cu	Mn	B	Reference
Ten locations, open-field	NA	NA	125.8 (122.3–130.8)	12.0 (11.2–12.5)	8.95 (7.66–10.06)	0.98 (0.72–1.17)	0.128 (0.113–0.142)	0.112 (0.093–0.141)	0.040 (0.028–0.053)	0.102 (0.065–0.140)	0.134 (0.109–0.156)	[33] ^1^
Ten locations, greenhouse	NA	NA	129.0 (125.3–133.8)	12.3 (12.0–12.6)	9.58 (8.45–11.02)	1.05 (0.80–1.25)	0.143 (0.125–0.164)	0.117 (0.098–0.152)	0.044 (0.033–0.057)	0.094 (0.061–0.133)	0.142 (0.116–0.170)	[33] ^1^
Ten varieties, open-field	NA	15.9 (12.4–21.2)	90.0 (68.4–117.7)	6.45 (4.00–9.55)	7.08 (5.97–11.35)	2.04 (1.44–2.67)	0.181 (0.111–0.239)	0.134 (0.081–0.182)	0.039 (0.023–0.058)	NA	NA	[14]
Seven varieties, open-field	NA	26.7 (22.9–34.4)	7.72 (4.08–10.38)	10.1 (7.0–13.3)	18.5 (14.8–23.0)	NA	0.145 (0.092–0.189)	NA	NA	NA	NA	[17] ^1^
Four varieties, open-field home gardens	NA	NA	179.3 (158–215)	9.4 (8.9–10.0)	5.13 (4.4–6.8)	1.51 (0.58–3.0)	0.313 (0.19–0.49)	0.191 (0.08–0.345)	0.117 (0.085–0.14)	0.035 (0.023–0.047)	NA	[28]
Two varieties, greenhouse, two different CO_2_ level	121.8 (101.4–147.6)	NA	27.6 (27.6–27.6)	9.9 (8.4–10.8)	8.4 (7.8–9.0)	NA	2.24 (2.06–2.33)	0.935 (0.795–1.178)	0.193 (0.167–0.214)	2.507 (2.388–2.589)	NA	[22] ^1^
Three varieties, greenhouse different N level, fungicide, degree of maturity	NA	13.4 (10.2–17.6)	230.4 (183.8–296.8)	10.1 (8.8–13.0)	5.05 (3.42–6.60)	5.06 (2.42–8.53)	0.46 (0.34–0.59)	0.14 (0.11–0.18)	0.13 (0.08–0.23)	NA	NA	[34]
Eight varieties, greenhouse	NA	18.4 (7.8–27.3)	284 (249–319)	16.5 (10.8–22.4)	15.0 (10.8–20.1)	6.7 (4.0–17.4)	1.67 (0.49–3.51)	1.79 (0.16–5.48)	0.19 (0.045–0.392)	0.18 (0.066–0.306)	NA	[25]
Twenty varieties, greenhouse	NA	NA	78.5 (75.9–81.0) ^2^	8.40 (7.88–8.92) ^2^	23.6 (22.7–24.4) ^2^	36.9 (35.4–38.4) ^2^	0.55 (0.52–0.58) ^2^	0.26 (0.25–0.27) ^2^	0.43 (0.41–0.45) ^2^	0.10 (0.10–0.10)^2^	NA	[27]
Ten wild tomato relatives, greenhouse	NA	NA	210.1 (194.3–222.5)	14.6 (12.9–20.5)	40.3 (37.7–46.9)	63.4 (54.9–84.4)	0.99 (0.96–1.17)	0.34 (0.17–0.70)	0.71 (0.62–0.74)	0.17 (0.15–0.19)	NA	[27]
Cherry variety Saopolo, greenhouse, different K:Ca fertilization, mature fruit	80.9 (71.4–91.6)	38.8 (17.5–53.9)	189.7 (149.1–232.7)	3.28 (1.50–4.74)	0.65 (0.60–0.78)	3.18 (0.90–4.62)	0.182 (0.149–0.222)	NA	0.051 (0.036–0.067)	NA	NA	[35] ^1^
Lycopersicon esculentum Mill. Open-field irrigation water salinity	NA	25.9 (23.4–30.6)	228.7 (202.2–263.4)	13.0 (11.4–15.6)	10.3 (7.8–15.0)	173.9 (45.0–300.0)	30.6 (26.2–37.0)	14.5 (13.0–16.6)	7.7 (6.4–9.2)	8.4 (7.0–10.6)	9.9 (8.8–11.4)	[29] ^1^

^1^ Values reported on a dry matter (DM) basis in the original studies were converted to a fresh matter (FM) basis assuming 94% moisture content. ^2^ Values are presented as mean with estimated range (mean ± SD). NA—not available.

## 5. Bioactive Compounds in Tomato Fruits

### 5.1. Carotenoids in Tomato Fruits

Tomatoes represent a significant dietary source of carotenoids, a class of bioactive compounds with well-documented antioxidant activity. These pigments are largely responsible for the characteristic red coloration of tomato fruits. Table 4 summarizes the carotenoid and ascorbic acid contents of tomato cultivars differing in fruit color and cultivation conditions. Lycopene, the main red pigment, is the predominant carotenoid in red tomatoes, typically occurring at concentrations of approximately 1–10 mg/100 g FM and accounting for about 80–90% of total carotenoids. β-Carotene is the second most abundant carotenoid (5–15%), followed by lutein (1–3%).

In orange tomatoes, β-carotene is the major pigment responsible for fruit coloration, ranging from up to 8.6 mg/100 g FM and representing approximately 15–25% of total carotenoids. Phytoene contributes around 80%, whereas lycopene accounts for only 10–15%. In yellow cultivars, the carotenoid profile is dominated by β-carotene, lutein, and early carotenoid precursors such as phytoene and phytofluene, whereas lycopene and lutein is present at rather low levels. Overall, yellow tomatoes contain markedly lower total carotenoid concentrations than red cultivars. Trace amounts of α-carotene and zeaxanthin have also been reported.

Carotenoid composition is strongly associated with fruit color. Red and pink cultivars generally contain higher carotenoid levels, whereas yellow and green cultivars are poorer sources [36]. Red cultivars predominantly accumulate lycopene, while orange cultivars are richer in β-carotene [37,38]. These differences reflect variation in carotenoid biosynthesis. In ripe tomato fruits, the activity of lycopene ε-cyclase (LCY-E) and lycopene β-cyclase (LCY-B), which convert lycopene into downstream carotenoids, is relatively low, allowing lycopene to accumulate as the dominant pigment. A key enzyme in this pathway is phytoene synthase (PSY), which catalyzes the formation of geranylgeranyl diphosphate (GGPP), the precursor of carotenoid biosynthesis [39,40,41].

In orange tomatoes, greater LCY-B activity promotes the conversion of lycopene to β-carotene. In yellow tomatoes, lycopene is often absent or present only at trace levels, likely because of an upstream limitation in carotenoid biosynthesis or rapid conversion into other carotenoids such as β-carotene and lutein. The accumulation of phytoene and phytofluene in these fruits supports this interpretation [40,42].

### 5.2. Environmental Factors Affecting Carotenoid Accumulation

Carotenoid accumulation is affected by several factors, including light intensity, temperature, cultivation system, and abiotic stress conditions. Light plays a major role in fruit coloration by regulating pathways involved in pigment synthesis. Increased light exposure during fruit development may significantly enhance carotenoid accumulation. For example, tomatoes grown in Spain, where the number of sunny days averages approximately 260–300 per year, contained about 75% more lycopene than those grown in Ireland, where sunshine duration is nearly half as high and total solar radiation is approximately 30% lower [43]. Red-light treatment has also been reported to increase lycopene accumulation, likely through the enhanced expression of phytoene synthase and stimulation of chloroplast-to-chromoplast transformation [44,45].

Temperature is a factor that affects lycopene synthesis. The optimal range for lycopene accumulation is approximately 12–32 °C, whereas temperatures outside this range may inhibit its formation [46]. Excessive heat or large temperature fluctuations may also induce physiological disorders such as yellow shoulder disorder, which reduces carotenoid accumulation in affected tissues and may lower lycopene content in tomato juice by 14–24% [47].

Cultivation conditions further modify carotenoid profiles. Tomatoes grown under open-fields have been reported to contain higher β-carotene but lower lycopene levels than greenhouse-grown fruits [48]. Mild abiotic stress may enhance carotenoid accumulation. For example, copper fertilization has been associated with increased lycopene content [49], and moderate salinity stress may raise the lycopene levels by up to 40% [29]. In addition, carotenoids are not uniformly distributed within the fruit: lycopene concentration is often three- to fivefold higher in the skin than in the pulp, whereas vitamin C is distributed more evenly [50].

**Table 4:** Carotenoids and vitamin C content (mg/100 g FM) from tomato fruit reported in the literature.

Varieties (Color, Type, Ripeness)	Cultivation Type, Country	Lycopene	β-Carotene	Lutein	Other Carotenoids	Total Carotenoids	Vitamin C	Reference
22 red, ripe, traditional	Greenhouse (net house), Spain	7.4 (1.3–14.1)	0.51 (0.35–1.00)	0.020 (0.012–0.032)	Phytoene 0.28 (0.17–0.38) Phytofluene 0.11 (0.04–0.19)	8.3 (2.1–15.6)	NA	[51]
8 red, mixed types, ripe, traditional	Open-field, organic, Spain	3.75 (2.54–6.23)	NA	NA	NA	3.75 (2.54–6.23)	27.2 (16.03–45.92)	[15]
5 red, cherry and round, ripe, post-harvested	Open-field, Costa Rica	0.78 (0.25–1.47)	10.4 (7.2–14.4)	NA	NA	11.2 (7.5–15.9)	67 (42–112)	[52] ^1^
7 red, ripe, regular	Greenhouse, Korea	2.27 (0.95–2.76)	0.75 (0.65–0.83)	NA	NA	3.02 (1.60–3.59)	9.6 (8.3–10.9)	[5]
13 red, ripe, cherry	Greenhouse, Korea	3.99 (2.02–5.12)	2.11 (0.98–3.56)	NA	NA	6.10 (3.00–8.68)	18.6 (9.7–22.5)	[5]
5 red (3 cherry, 2 common), ripe	Greenhouse, Spain	4.19 (0.28–7.03)	0.35 (0.03–0.97)	0.12 (0.03–0.22)	Phytoene 1.02 (0.49–1.63) Phytofluene 0.06 (0.00–0.14)	6.37 (0.86–9.98)	NA	[37] ^1^
43 red, ripe, cherry, regular varieties and breeding lines	Greenhouse, Korea	4.73 (3.10–12.20)	0.50 (0.24–1.04)	0.17 (0.05–0.40)	NA	5.43 (3.49–12.30)	11.7 (1.07–32.75)	[53] ^1^
76 red, ripe, non-cherry, regular varieties and breeding lines	Greenhouse, Korea	5.34 (2.32–12.40)	0.43 (0.18–1.04)	0.14 (0.07–0.31)	NA	6.09 (3.12–12.95)	14.3 (4.30–38.80)	[53] ^1^
3 red, ripe	Greenhouse, differed nitro-gen fertilization Spain	4.26 (2.60–6.58)	0.57 (0.35–0.77)	0.19 (0.10–0.28)	Phytoene 0.82 (0.48–1.21) Phytofluene 0.12 (0.06–0.21)	5.97 (4.06–8.62)	30.0 (24.9–36.3)	[34]
6 red, ripe, cherry	Open-field, India	8.75 (5.20–15.10)	1.72 (0.80–3.02)	NA	NA	14.87 (10.70–23.80)	NA	[38] ^1^
4 red, ripe, commercial	NA	2.38 (1.47–3.17)	0.38 (0.23–0.49)	0.031 (0.017–0.045)	NA	2.79 (1.72–3.70)	NA	[43] ^1^
4 red, ripe, commercial	NA	4.14 (1.81–7.96)	0.44 (0.35–0.58)	0.119 (0.081–0.183)	NA	4.70 (2.24–8.73)	NA	[43] ^1^
1 red, ripe, hybrid HC01	Open-field, different salinity and nitrogen fertilization, Italy	7.80 (5.89–10.22)	0.35 (0.31–044)	NA	Phytoene 0.72 (0.63–0.86) Phytofluene 0.52 (0.45–0.62) ζ-Carotene 0.09 (0.06–0.11)	9.49 (7.37–12.26)	0.61 (0.54–0.68)	[29]
26 red and pink (including red-green, red with green streaks)	Open-field, Romania (local varieties collected from several countries)	6.45 (0.07–12.93)	0.89 (0.01–2.09)	0.37 (0–0.87)	NA	7.71 (0.08–14.03)	NA	[54]
12 pink and pink-black, ripe, traditional varieties	Greenhouse (net house), Spain	6.1 (1.6–12.7)	0.37 (0.26–0.48)	0.015 (0.009–0.019)	Phytoene 0.31 (0.17–0.38) Phytofluene 0.09 (0.04–0.14)	6.9 (2.2–13.6)	NA	[51]
8 red-black, ripe traditional	Greenhouse (net house), Spain	7.9 (4.0–14.1)	0.55 (0.35–0.90)	0.042 (0.015–0.103)	Phytoene 0.26 (0.13–0.59) Phytofluene 0.094 (0.047–0.187)	8.85 (5.2–16.0)	NA	[51]
9 orange and yellow, ripe	Open-field, Romania (local varieties collected from several countries)	2.61 (0.09–12.93)	1.28 (0.10–6.55)	0.25 (0.14–0.38)	NA	4.13 (0.33–14.01)	NA	[54]
2 orange (cherry and common), ripe	Greenhouse, Spain	1.5 (0.0–3.1)	0.12 (0.05–0.19)	0.05 (0.04–0.06)	Phytoene 13.9 (2.5–25.3) Phytofluene 0.78 (0.32–1.23)	16.4 (3.0–29.8)	NA	[37] ^1^
3 orange, cherry, ripe	Open-field, India	2.05 (0.85–3.30)	6.88 (5.65–8.56)	NA	NA	9.15 (8.20–9.74)	NA	[38]
1 yellow, ripe	Greenhouse, Spain	NA	0.12 (0.12–0.12)	0.10 (0.10–0.10)	Phytoene 0.03 (0.03–0.03)	0.25 (0.25–0.25)	NA	[37] ^1^
11 yellow and yellow-black, ripe traditional	Greenhouse (net house), Spain	0.049 (0–0.24)	0.10 (0.04–0.16)	0.019 (0.009–0.031)	Phytoene 0.036 (0.017–0.043)	0.21 (0.08–0.32)	NA	[51]
3 green, ripe	Greenhouse, Spain	1.85 (0.00–4.65)	0.25 (0.07–0.41)	0.22 (0.05–0.37)	Phytoene 1.39 (0.70–2.75)	3.76 (2.13–6.12)	NA	[37] ^1^
High-lycopene tomato genotypes (red ripe stage)	Greenhouse, USA	9.24 (4.65–15.36)	0.41 (0.06–1.83)	NA	Phytofluene 0.17 (0.03–0.6)	9.82 (4.74–17.80)	NA	[55]

^1^ Values reported on a dry matter (DM) basis in the original studies were converted to a fresh matter (FM) basis assuming 94% moisture content. NA—not available.

### 5.3. Lycopene: Structure and Biological Importance

Tomatoes are the main dietary source of lycopene, a carotenoid belonging to the terpenoid group of natural pigments. Unlike α-carotene and β-carotene, lycopene does not exhibit provitamin A activity because it lacks a terminal β-ionone ring. Nevertheless, it is a potent antioxidant and plays an important role in protection against oxidative stress [56].

Chemically, lycopene is an unsaturated hydrocarbon composed of 40 carbon atoms with 11 conjugated and 2 non-conjugated double bonds. In natural products, it occurs mainly in the all-trans form, although cis-isomers of lycopene predominate in human tissues and plasma. Cis-lycopene isomers are also preferentially absorbed in the small intestine from dietary sources likely due to their greater solubility in mixed micelles and lower tendency to aggregate. Exposure to heat, oxygen, or light may induce isomerization to cis forms. This helps explain why processed tomato products often provide more bioavailable lycopene than raw tomatoes [57,58].

There are several reviews and meta-analyses discussing the biological activity of lycopene and its potential role in disease prevention. Ref. [59] analyzed data concerning the role of lycopene in lung cancer prevention. The proposed mechanisms include the inhibition of cancer cell proliferation, angiogenesis, and metastasis, as well as the induction of apoptosis through the modulation of cellular redox status, regulation of growth factor signaling pathways, alterations in cell growth-related enzymes, and enhancement of gap-junction communication. Animal studies have shown that lycopene is mainly accumulated and stored in the liver, although significant amounts can also be found in other organs. Epidemiological studies suggest an association between lycopene intake and a reduced incidence of lung cancer. However, these findings should be interpreted with caution, as higher lycopene intake may simply reflect a greater consumption of fruits and vegetables and an overall healthier lifestyle.

Lycopene may also positively affect bone metabolism by promoting osteoblast differentiation, stimulating collagen production, and suppressing key proteins involved in bone resorption. In a pilot clinical study involving postmenopausal women, the consumption of lycopene-rich tomato sauce was associated with the prevention of bone mineral density loss compared with the control group. However, it should be noted that this was a pilot study with a relatively small cohort and a short intervention period of only three months [60].

In a randomized crossover study involving healthy postmenopausal women, daily consumption of tomato purée increased the plasma lycopene concentrations by approximately 40% after 24 h and by about 150% after 7 days. Despite this marked increase in circulating lycopene levels, no improvement in endothelial function assessed by flow-mediated dilation (FMD) was observed [61].

On the other hand, Ref. [62] reported reductions in total and LDL cholesterol levels after four weeks of tomato juice consumption in adults with stage 1 hypertension. Interestingly, fortification of the juice with a polyphenol-rich ethanolic extract did not provide any additional benefit. This observation may suggest that compounds naturally present in tomato products, including lycopene and other carotenoids, could contribute to the observed effects on cardiovascular risk markers, although their individual contributions remain unclear. While current findings indicate a promising role of lycopene in disease prevention and health promotion, the available human evidence remains insufficient to support specific intake recommendations. Although epidemiological studies suggest beneficial associations between lycopene consumption and health outcomes, these observations may be influenced by confounding dietary and lifestyle factors. Therefore, additional long-term, well-controlled intervention studies are necessary to establish causal relationships and clarify the biological mechanisms involved [63,64,65,66].

### 5.4. Bioaccessibility and Processing Effects of Carotenoids

Carotenoid bioaccessibility depends strongly on chemical structure. Although lycopene is the dominant carotenoid in tomatoes, its bioaccessibility is relatively low and usually does not exceed 1%. β-Carotene is somewhat more bioaccessible, whereas lutein may reach much higher values due to the presence of hydroxyl groups that increase its polarity and facilitate micellar transfer during digestion [43].

Carotenoid bioaccessibility is strongly influenced by the plant matrix from which these compounds are released, as well as by other dietary components present in the meal. Because carotenoids are lipophilic molecules, their absorption is enhanced by the presence of dietary fat. Before micellarization can occur, carotenoids must first be released from the tomato matrix and transferred into the lipid phase, making this step a critical determinant of their bioaccessibility. Ref. [67] demonstrated that even in the presence of a large excess of oil, only about 30% of lycopene was transferred into the lipid phase, indicating that the food matrix itself constitutes a major barrier to carotenoid release. Furthermore, the presence of emulsifiers, low temperature, or a high dietary fiber content may reduce carotenoid bioaccessibility by limiting their release and incorporation into mixed micelles [68]. Enzymatic treatments that disrupt cell wall polysaccharides have therefore been proposed as a strategy to improve carotenoid release and subsequent bioaccessibility [69].

Processing may substantially improve carotenoid bioavailability. Chopping, grinding, and homogenization disrupt plant tissues and release carotenoids from the food matrix, while heating increases extractability by breaking protein–carotenoid complexes [70,71].

Heating tomato slurry at 88 °C for 30 min has been reported to double the extractable lycopene compared with fresh tomatoes [72]. Similarly, industrial tomato paste production retains most of the original lycopene, although β-carotene losses of about 30% may occur [73]. In contrast, drying may result in greater carotenoid degradation, especially at temperatures above 70–80 °C, due to oxidation, double-bond cleavage, and isomerization [74,75]. Lycopene is generally more stable than β-carotene during processing, but even freeze-drying may lead to substantial losses because of tissue disruption and increased oxygen exposure [42].

Thermal sterilization promotes the conversion of lycopene into cis-isomers, which are believed to have higher bioavailability. However, such treatments may also cause substantial carotenoid losses due to oxidative degradation. Recently, there has been increasing interest in non-thermal processing technologies. One of the most widely used methods is high-pressure processing (HPP). Although HPP alone may not be fully effective in reducing bacterial spores or inactivating some enzymes, when combined with a short heat treatment, such as blanching, it can help protect tomato products from spoilage during long-term storage. Compared with thermal sterilization, HPP better preserves lycopene integrity by limiting its degradation and isomerization. This treatment can also improve the color of tomato juice. However, its effect on lycopene bioaccessibility is limited, and lycopene bioaccessibility remains low [76,77,78,79].

The cell walls of tomato tissues appear to be an important factor limiting carotenoid transfer. Therefore, high-pressure homogenization (HPH) can enhance carotenoid release from the plant matrix without inducing significant chemical modifications of these compounds. In tomato juice production, HPH treatment reduced particle diameter by approximately 20-fold, resulting in improved uniformity, increased viscosity, and enhanced color attributes [79,80].

Pulsed electric field (PEF) treatment enhances the permeability of plant cell membranes through electroporation, facilitating the release of carotenoids from tomato tissues. However, the effect of PEF on carotenoid bioaccessibility requires further investigation. While [81] reported a substantial increase in lycopene bioaccessibility following PEF treatment of whole tomatoes, Ref. [82] observed no improvement, and in some cases, even a decrease, in carotenoid bioaccessibility in isolated chromoplast fractions.

The effects of pulsed light (PL) and UV-C treatments on tomatoes could also be interesting. These treatments may be effective in reducing surface contamination and increasing tomato safety without the deterioration of other quality parameters. Interestingly, in the case of immature tomatoes, treatment with PL or UV-C caused a significant increase in carotenoid content after storage. The visible spectrum seems to be more effective than UV light, as excessive exposure to UV may inactivate enzymes responsible for lycopene synthesis. The lycopene content in treated samples could be even five times higher than in untreated samples. This suggests that these treatments could be useful post-harvest methods for immature tomato fruits to enhance lycopene accumulation during storage [83,84,85].

Tomato processing by-products, particularly peels, seeds, and residual pulp, can still be a rich source of bioactive compounds, mainly carotenoids and polyphenols. These by-products can be easily dried and milled into powder, which may then be incorporated into other food products to improve their nutritional value and antioxidant potential. Carotenoids can also be effectively extracted using organic solvents. The obtained extracts may be stabilized through microencapsulation with wall materials such as maltodextrin combined with gum Arabic or whey protein. Microencapsulation has been shown to effectively protect carotenoids against degradation during processing and storage. However, despite improved stability, the fraction of carotenoids that becomes potentially available for absorption after *in vitro* digestion remains relatively low, reaching only about 25% of the initial content. This highlights the need for further optimization of encapsulation systems, not only to improve carotenoid stability but also to enhance their bioaccessibility [86,87].

Another approach to stabilizing carotenoid extracts is the development of nanoemulsions using edible oils as the lipid phase. Such nanoemulsions exhibit high storage stability and can serve as effective ingredients in the formulation of functional beverages. The fatty acid composition of the lipid phase plays a crucial role in micelle formation during digestion, and consequently, in the release and potential absorption of lycopene. In the case of nanoemulsions prepared with olive pomace oil, lycopene bioaccessibility may reach up to 50%, highlighting the potential of these delivery systems to enhance the nutritional value of carotenoid-rich products [88].

**Figure 1:**
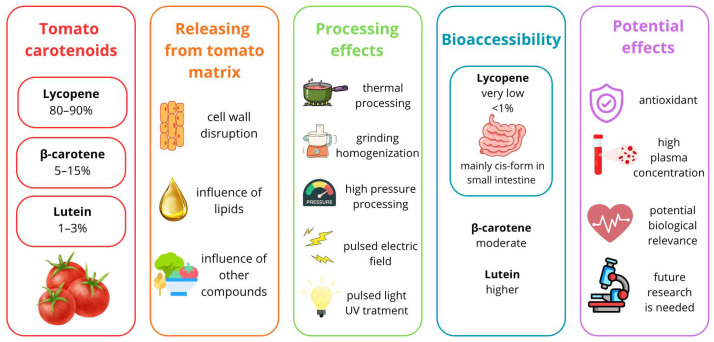
Overview of tomato carotenoids, their release from the food matrix, processing effects, bioaccessibility, and potential biological relevance.

Overall, many processing technologies can improve carotenoid extractability and stability. However, this does not necessarily translate into high bioaccessibility. Further research is therefore needed to optimize processing conditions and delivery strategies that balance carotenoid stability, product quality, and actual bioaccessibility (Figure 1).

### 5.5. Ascorbic Acid Content

Plants from the Solanaceae family are an important dietary source of vitamin C. For example, bell peppers contain 80–200 mg/100 g FM, which may be up to five times higher than in citrus fruits. Tomato fruits contain lower amounts but still represent a relevant source of ascorbic acid, with reported levels ranging from 12 to 48 mg/100 g FM and an average value of approximately 24 mg/100 g FM (Table 4). Only one study reported a markedly lower content, below 1 mg/100 g FM. However, this discrepancy may be explained not only by varietal and environmental differences, but also by the analytical method used, as ascorbic acid was measured only in juice squeezed from the fruits [29].

Data presented in Table 4 indicate considerable genotypic variation in ascorbic acid content. Fruit size may also influence vitamin C levels, as small-fruited cherry-type cultivars are often characterized by higher concentrations. Although carotenoids and ascorbic acid are synthesized through different metabolic pathways, some relationship between these antioxidant compounds has been observed. Based on the mean values presented in Table 4, carotenoid and vitamin C contents appeared to be moderately positively associated (*n* = 8, *r* ≈ 0.65).

Similarly to carotenoids, vitamin C synthesis may be influenced by mild stress during fruit development. For example, Ref. [29] reported that increasing salinity levels resulted in a nearly linear increase in ascorbic acid concentration in tomato fruits. Ascorbate oxidase, an enzyme catalyzing the oxidation of ascorbic acid, is present in tomato fruits. Its activity varies among breeding lines. Tomato lines with lower ascorbate oxidase activity were found to contain higher levels of ascorbic acid. This effect was particularly evident when plants were grown under moderate and high salinity conditions [89].

The content of ascorbic acid also changes during fruit maturation. Green, unripe tomatoes contain at least 10% less vitamin C than fruits at the red-ripe stage. At full ripeness, vitamin C levels generally reach a plateau or decrease slightly [90,91].

Because vitamin C is relatively unstable, significant losses may occur during processing. High temperatures accelerate its degradation, particularly with prolonged heating. For example, Ref. [72] reported that heating tomato slurry at 88 °C for only 2 min resulted in a 10% loss of ascorbic acid, whereas extending the heating time to 30 min increased the losses to approximately 30%. Processed tomato products such as juice, purée, and paste contain about 12%, 22%, and 36% less vitamin C, respectively, than fresh fruits [92].

Even non-thermal processing can cause losses of ascorbic acid. However, the level of degradation is usually lower and strongly depends on the processing conditions. Ref. [93] reported that HHP treatment of tomato juice resulted in up to 50% degradation of ascorbic acid, whereas [94] observed only a 2% decrease. Vitamin C also degrades during storage. In addition to chemical oxidation, enzymatic activity may contribute to this process. Ref. [94] showed that polyphenol oxidase (PPO) and peroxidase (POD) were only partially inactivated by HHP, retaining approximately 98% and 81% of their initial activity, respectively.

Ascorbic acid also degrades during drying. Its degradation kinetics, similar to carotenoids, follow a first-order reaction model. However, under low-temperature drying conditions, vitamin C may be better preserved than β-carotene. For instance, during solar drying at 40–65 °C, vitamin C losses were approximately 30%, whereas β-carotene degradation reached about 80% [74,75].

As tomatoes are a moderate source of vitamin C, the selection of cultivars with a high ascorbic acid content and low activity of ascorbic acid-degrading enzymes may be beneficial. Furthermore, because ascorbic acid is highly unstable and substantial losses may occur during postharvest handling, storage, and processing, all of these conditions should be carefully optimized to maximize its retention in tomato products.

### 5.6. Polyphenols in Tomatoes

Phenolic compounds are among the key secondary metabolites responsible for the biological and health-promoting properties of tomatoes. The polyphenol profile of tomato fruits is highly diverse. Numerous studies have identified and quantified phenolic compounds using analytical methods ranging from the Folin–Ciocalteu assay to advanced techniques such as HPLC-MS. The phenolic profile of different tomato cultivars is summarized in Table 5.

More than 60 individual phenolic compounds have been reported in tomatoes, which can be classified into six main subclasses. The most abundant groups are hydroxycinnamic acids and their derivatives, including caffeic, chlorogenic, p-coumaric, and ferulic acids, together with their isomers and conjugated forms. Another important subgroup is flavonols, including rutin, quercetin, kaempferol, and their glycosylated derivatives. Flavanones, chalcones, and dihydrochalcones form another class, mainly represented by naringenin and its derivatives. Hydroxybenzoic acids such as protocatechuic, gentisic, and benzoic acids and their glycosides have also been identified. In addition, compounds such as homovanillic and homoveratric acids, as well as benzyl alcohol derivatives, have been reported [95,96].

Table 6 and Table 7 summarize the quantitative data on phenolic compounds reported in the literature. Total polyphenol content (TPC), determined by the Folin–Ciocalteu method, varies widely, ranging from 5 to 217 mg/100 g FM (Table 6). This variation may result from differences in cultivar, growing conditions, and analytical procedures. In particular, methodological factors such as extraction conditions, extraction solvents, and assay protocols can substantially influence the reported values. Table 7 presents the concentrations of individual phenolic compounds determined mainly by HPLC.

TPC values obtained using the Folin–Ciocalteu method are generally higher than the sum of individual phenolics quantified by HPLC, and the correlation between these two approaches is often weak. This discrepancy is mainly due to the limited specificity of the Folin–Ciocalteu reagent, which reacts not only with phenolic compounds but also with other reducing substances naturally present in tomatoes. As a result, TPC may be overestimated. Moreover, individual phenolic compounds differ in their response to the reagent, while results are expressed as gallic acid equivalents.

Among the phenolic groups present in tomatoes, hydroxycinnamic acids occur at the highest levels (Table 7). The main representatives include ferulic, caffeic, p-coumaric, and chlorogenic acids and their derivatives. In contrast, hydroxybenzoic acids occur at much lower concentrations; for example, gallic acid and its derivatives rarely exceed 3.36 mg/100 g FM. The second most abundant group comprises flavonols and their glycosides, with rutin often reported as the dominant individual compound. Quercetin and its derivatives are also frequently identified in tomato fruits. Another important group includes flavanones and chalcones, mainly naringenin derivatives, with reported concentrations ranging from 0.05 to 27.98 mg/100 g FM. Minor phenolic compounds detected in tomatoes include phloretin, phloridzin, luteolin, and their derivatives, which have been reported only in a few studies and generally at low concentrations.

Studies investigating the biological activity of tomato polyphenols have demonstrated various bioactive effects. In cell culture experiments conducted by [18], tomato extracts stimulated the growth of normal liver cells (Chang) and had little effect on normal lung cells (Hel299). The extracts produced only a mild inhibition of lung cancer cells (A549), whereas in lymphoma cells (U937), they initially stimulated growth and inhibited it at higher concentrations. The authors reported no clear relationship between the measured chemical composition and the observed effects on cell proliferation.

Ethanolic extracts of tomato have also demonstrated antimicrobial activity against several bacterial species, including *Staphylococcus aureus*, *Bacillus subtilis*, *Escherichia coli*, and *Pseudomonas aeruginosa* [52].

### 5.7. Changes in Polyphenols During Ripening and Processing

The polyphenolic profile and content of tomatoes change during fruit ripening. Generally, tomatoes at the green stage contain a more diverse range of phenolic compounds than fully ripe fruits. In unripe tomatoes, in addition to phenolic acids and flavonols, phenolics are mainly represented by flavanols such as catechin and epicatechin, as well as oligomeric procyanidins (B1, B2, B3, and C1). During ripening, these compounds are metabolized and hydrolyzed into smaller molecules. At the same time, tomatoes begin to accumulate relatively higher levels of phenolic acids, including chlorogenic and caffeic acids and their derivatives, as well as p-coumaric acid derivatives and flavonols such as rutin [103,104].

Fertilization during tomato cultivation can significantly influence the polyphenol content, although the effect depends on fertilizer composition. Increasing nitrogen fertilization from 0 to 200 kg/ha increased the lycopene and soluble polyphenol contents but decreased the total flavonoids, ascorbic acid, and antiradical activity. A moderate positive correlation (r = 0.52) between nitrogen fertilization and soluble polyphenol content has been reported. However, the effect on polyphenol levels was relatively moderate, with increases of approximately 2–38% compared with the non-fertilized control, and it varied depending on cultivation year and cultivar [97]. A stronger effect has been observed with NPK fertilization. Increasing NPK doses from 40 to 120 kg/ha nearly doubled the polyphenol content. Moreover, the use of salicylic acid in fertigation significantly increased phenolic compound synthesis, likely through activation of the phenylpropanoid pathway [98].

The biosynthesis of phenolic compounds in plants is strongly influenced by biotic and abiotic stress factors, which can affect the health-promoting properties of tomato fruits. For example, flavonols such as quercetin and kaempferol may be produced in response to mechanical damage or pathogen attack, whereas chlorogenic acid and other phenolic esters, whose synthesis increases after tissue injury, act as defensive compounds [105].

Processing conditions may also affect the stability of phenolic compounds. High hydrostatic pressure processing of tomato juice has been shown to influence certain phenolics, particularly hydroxycinnamic acids. For example, treatment at 550 MPa for 10 min reduced the levels of p-coumaric, ferulic, and caffeic acids by 73%, 33%, and 14%, respectively, whereas chlorogenic acid derivatives showed greater stability, with losses of cryptochlorogenic acid not exceeding 10%. In comparison, phenolic compounds appeared more stable during thermal processing. Heating tomato juice at 110 °C for approximately 9 s did not reduce the total phenolic content and even increased the concentration of some compounds [106]. Interestingly, during refrigerated storage, the concentrations of some phenolic compounds increased. This phenomenon was observed for caffeic acid, quercetin, ferulic acid, and p-coumaric acid in both HHP- and thermally treated tomato juice. This increase may be attributed to the release of phenolic compounds from bound forms. The effect was more evident after HHP treatment, where many endogenous enzymes remain active [94].

### 5.8. Tocopherols from Tomatoes Fruit

Although tomatoes do not contain the highest levels of vitamin E among other sources, their widespread consumption worldwide makes them an important dietary source of this vitamin. Tomato fruits contain several tocopherol isomers. The main forms reported in the literature include α-tocopherol, α-tocopherol quinone, α-tocopherol fatty acid ester, α-tocopherol hydroquinone, γ-tocopherol, and β-tocopherol. In some studies, β-tocopherol was detected chromatographically but not quantified (Table 8) [107,108,109].

The concentration of tocopherols in tomatoes is strongly influenced by environmental conditions at the cultivation site. Higher temperatures and lower rainfall have been associated with increased tocopherol levels. In addition, vitamin E content may vary depending on abiotic stresses such as low temperature, high temperature, and salinity. Cultivar also plays an important role, with smaller-fruited tomatoes often showing higher tocopherol concentrations. Because seeds are a major source of tocopherols, tomato processing by-products such as pomace, consisting mainly of skins and seeds remaining after juice extraction, may contain relatively high tocopherol levels [107,108,110].

Nutrient availability in soil may also influence vitamin E accumulation. For example, Ref. [109] suggested that potassium fertilization may promote tocopherol biosynthesis, as a positive correlation between potassium content and vitamin E levels in tomatoes was observed. Genetic factors also contribute to vitamin E variation. A major quantitative trait locus affecting vitamin E content in tomato fruits (mQTL9-2-6) has been mapped to the VTE3(1) gene, which encodes the enzyme 2-methyl-6-phytylquinol methyltransferase involved in the biosynthesis of α- and γ-tocopherol. Furthermore, this QTL appears to be epigenetically regulated. Differences in vitamin E levels have been associated with DNA methylation patterns in the promoter region of VTE3(1), which influence gene expression [111].

## 6. Potential Health Risks and Antinutritional Compounds in Tomatoes

In addition to compounds with beneficial health effects, tomatoes also contain substances that may raise certain health concerns. Among these are glycoalkaloids, biogenic amines, and lectins, which may exhibit toxic or antinutritional effects under specific conditions.

One of the main glycoalkaloids present in tomatoes is α-tomatine, a compound responsible for the bitter taste of unripe fruits. It occurs in relatively high concentrations in green, immature tomatoes as well as in other parts of the plant, including leaves, flowers, stems, and roots. In immature fruits, α-tomatine acts as a natural defense compound protecting the plant against pathogens, fungi, insects, and herbivores. During fruit maturation and ripening, the concentration of α-tomatine decreases dramatically, to almost no detectable level. This glycoalkaloid is metabolized into esculeoside A, a less toxic and non-bitter compound (Table 9). The reduction in glycoalkaloid content in ripe fruits may facilitate consumption by frugivores and consequently promote seed dispersal. This mechanism differs from that observed in potatoes, another commonly consumed *Solanaceae* plant. In potatoes, glycoalkaloid levels remain relatively high because the edible organ is the storage tuber, which requires protection from herbivores [112,113,114,115].

Another group of compounds occasionally present in tomatoes is biogenic amines. These low-molecular-weight compounds may cause adverse health effects when present at high concentrations in food, and their presence may also indicate microbial spoilage. In a study conducted by [116], ten biogenic amines were analyzed in tomato products: methylamine, ethylamine, putrescine, cadaverine, histamine, spermidine, spermine, tyramine, phenylethylamine, and tryptamine. The total biogenic amine content was reported to be 7.11 mg/L in fresh tomatoes, 5.12 mg/L in canned chopped tomatoes, and 7.69 mg/L in tomato pulp. Histamine and tyramine are considered the most toxicologically relevant biogenic amines. According to the EFSA, no adverse health effects have been observed in healthy individuals after the consumption of up to 50 mg of histamine and 600 mg of tyramine per meal. In contrast, no safety thresholds have been established for putrescine and cadaverine [117]. The concentrations detected in tomato samples were substantially lower than these levels.

Tomatoes also contain lectins, which are carbohydrate-binding proteins capable of interacting with the gastrointestinal tract. Tomato lectins may bind to intestinal epithelial cells, exhibit bioadhesive properties, and interact with mucus. They can also be transported across enterocytes to gut-associated lymphoid tissue, potentially influencing mucosal barrier function and immune responses. In addition, tomato lectins demonstrate hemagglutinating activity toward erythrocytes and mitogenic activity toward immune cells, suggesting possible immunomodulatory properties. *In vitro* studies have shown that purified tomato lectin stimulated the proliferation of mouse splenocytes in a dose-dependent manner up to a concentration of 62.5 μg/mL (0.8 mM). At higher concentrations, the mitogenic response decreased progressively [118,119,120].

Overall, although tomatoes contain several compounds with potential toxic or antinutritional properties, their concentrations in ripe fruits and processed products are generally low and do not pose significant health risks under normal dietary consumption.

## 7. Summary and Future Perspective

Tomatoes are plant raw material of high nutritional and functional value, and their fruits represent an important source of vitamins, minerals, and numerous bioactive compounds, including carotenoids, polyphenols, ascorbic acid, and tocopherols. The content of these compounds depends on several factors, such as cultivar, fruit ripeness, environmental conditions, and the cultivation and processing methods applied, which means that their health-promoting properties can be optimized. Carotenoids, particularly lycopene, are responsible for the characteristic color of tomato fruits and exhibit strong antioxidant properties. Polyphenols, including rutin and chlorogenic acid, together with tocopherols, enhance the antioxidant capacity of tomatoes by protecting cellular components and lipids from oxidation, whereas glycoalkaloids such as tomatine play a protective role in the plant. The available evidence suggests that the regular consumption of tomatoes and tomato products may be associated with potential health benefits, including support of normal body functions and a possible reduction in the risk of cancer.

From a future perspective, further research should focus on the identification and selection of breeding lines characterized by high concentrations of bioactive compounds and reduced enzymatic activity that may contribute to their degradation. In addition, greater attention should be given to the optimization of postharvest treatments, as these may significantly influence the nutritional quality of tomatoes. Further studies are also needed to improve processing technologies, particularly non-thermal methods, with the aim of achieving an optimal balance between sensory attributes and the preservation of nutritional value.

Although epidemiological studies suggest beneficial associations between lycopene intake and various health outcomes, these observations may be influenced by confounding dietary, environmental, and lifestyle factors. Therefore, additional long-term, well-controlled intervention studies are needed to establish causal relationships and to clarify the underlying biological mechanisms. Such evidence would provide a stronger scientific basis for considering tomatoes and their bioactive constituents as functional foods with potential health-promoting properties.

## Figures and Tables

**Table 5:** Phenolic compounds identified in tomato fruit [95,96].

Phenolic Class	Representative Compounds
Chlorogenic acids (Caffeoylquinic acids)	Chlorogenic acid (3-CQA), Neochlorogenic acid, 1-CQA, 4-CQA, 5-CQA, Dicaffeoylquinic acid (isomers I, II, III, 1,5-, 3,5-, 4,5-), Tricaffeoylquinic acid (1,3,5-TQA), and 3-Caffeoylshikimic acid.
Hydroxycinnamic acid derivatives	Caffeic acid, p-Coumaric acid, Ferulic acid, Isoferulic acid, Sinapic acid hexose, Caffeoyl-hexose (isomers I-IV), Caffeoyl glucoside, Feruloyl-hexose, Coumaroyl-hexose, Dihydrocaffeic acid-3-O-glucoside, and Dihydrosinapic acid-4-O-glucoside.
Flavonols and their glycosides	Rutin (Quercetin-3-rutinoside), Quercetin, Quercetin-3-O-glucoside, Quercetin-5-sophoroside, Quercetin-3-O-arabinoside-5-O-rutinoside, Rutin hexoside, Rutin pentoside, Kaempferol-3-rutinoside, Kaempferol-3,7-dihexoside, and Isorhamnetin derivatives.
Flavanones, Chalcones, and Dihydrochalcones	Naringenin, Naringenin-7-O-glucoside, Naringenin-5-O-glucoside, Naringenin-7-O-galactoside, Naringenin-7-O-arabinoside, Naringenin-4′-O-glucoside, Naringenin chalcone, Eriodictyol, Eriodictyol chalcone, and Phloretin dihexoside.
Hydroxybenzoic acids and derivatives	Protocatechuic acid, Gentisic acid, Benzoic acid, p-Hydroxybenzaldehyde, Hydroxybenzoic acid hexose, and Dihydroxybenzoic acid pentose.
Phenylacetic acids and other phenolic compounds	Homovanillic acid hexose (isomers I, II, III), Homoveratric acid, Apigenin acetylhexoside, Benzyl alcohol derivatives, and 3,4-Dihydroxyphenyl-1-methyl ester-carbamic acid.

**Table 6:** Total phenolic content (TPC) of tomato fruits determined by the Folin–Ciocalteu method reported in the literature.

Total Polyphenol Content TPC mg/100 g FM Mean (Min–Max)	Reference
12.7 (10.1–17.4) ^1^	[5]
16.0 (15.4–16.6)	[42]
23.6 (17.8–30.5)	[97]
24 (5–42) ^1^	[36]
29.6 (19.2–39.8) ^1^	[18]
29.9 (19.2–39.6)1	[52]
38 (22–49)	[14]
62.4 (37.3–80.3)	[98]
126.7 (79.7–190.7) ^1^	[53]
129 (90–217) ^1^	[99]
159.2 (126.0–187.2) ^1^	[100]
170 (126–204) ^1^	[101]

^1^ Values reported on a dry matter (DM) basis in the original studies were converted to a fresh matter (FM) basis assuming 94% moisture content.

**Table 7:** Quantitative determination of main phenolic compounds found in red ripe tomato fruit reported in the literature.

Phenolic Compounds/Group	Class of Phenolic Compounds	Mean (Min–Max) mg/100 g FM	Reference
Caffeic acid and derivatives	Hydroxycinnamic acids	21.8 (3.46–45.6) ^1^	[99]
		5.13 (3.9–6.4)	[102]
		4.01 (3.45–4.90) ^1^	[95]
		0.86 (0.23–1.81) ^1^	[37]
		0.74 (0.34–1.12) ^1^	[18]
		0.70 (0.47–0.93)	[96]
Coumaric acid and derivatives	Hydroxycinnamic acids	21.5 (6.1–46.6) ^1^	[99]
		4.47 (3.66–5.87) ^1^	[101]
		1.93 (0.34–6.24) ^1^	[37]
		1.60 (1.43–1.85) ^1^	[95]
		0.21 (0.05–0.37)	[102]
		0.18 (0.03–0.28)	[96]
Chlorogenic acid and derivatives (caffeoylquinic acids)	Hydroxycinnamic acid	16.0 (3.9–46.5) ^1^	[99]
		3.75 (2.91–4.45) ^1^	[95]
		3.67 (0.74–7.30) ^1^	[18]
		2.48 (0.23–5.10) ^1^	[37]
		2.03 (0.81–5.60)	[14]
		1.86 (1.45–2.08)	[96]
		1.76 (0.90–2.53) ^1^	[101]
Ferulic acid and derivatives	Hydroxycinnamic acids	36.7 (12.2–52.5) ^1^	[99]
		7.46 (4.70–9.41) ^1^	[101]
		0.86 (0.77–0.94) ^1^	[95]
		0.80 (0.55–0.93) ^1^	[37]
		0.08 (0.01–0.22)	[96]
		0.04 (0.02–0.06)	[102]
Gallic acid and derivatives	Hydroxybenzoic acid	2.90 (2.55–3.36) ^1^	[101]
		0.55 (0.47–1.30) ^1^	[37]
Protocatechuic acid and derivatives	Hydroxybenzoic acid	1.13 (0.47–2.21) ^1^	[95]
		0.60 (0.14–1.00) ^1^	[99]
Quercetin and derivatives	Flavonol	6.45 (5.16–7.44) ^1^	[5]
		5.33 (3.70–7.27) ^1^	[53]
		2.46 (0.50–6.73) ^1^	[18]
		2.38 (1.34–3.73) ^1^	[37]
		2.16 (0.15–6.18)	[101]
		2.02 (1.34–3.90) ^1^	[99]
		1.00 (0.78–1.22)	[102]
		0.53 (0.44–0.67)	[96]
		0.04 (0.02–0.06) ^1^	[95]
Rutin and derivatives	Flavonol glycoside	42.6 (11.0–85.5) ^1^	[99]
		5.05 (3.69–6.41)	[102]
		4.68 (3.06–6.42) ^1^	[5]
		4.12 (2.25–8.02)	[14]
		2.48 (0.79–3.77) ^1^	[101]
		1.94 (1.32–3.24) ^1^	[53]
		1.81 (0.22–5.33) ^1^	[18]
		0.85 (0.57–1.19) ^1^	[95]
		0.59 (0.43–0.70)	[96]
Naringenin and derivatives	Flavanones/ chalcones	12.1 (0.0–27.5) ^1^	[18]
		9.60 (0.61–27.98) ^1^	[99]
		4.87 (3.72–6.02)	[102]
		3.47 (0.60–11.02) ^1^	[101]
		2.51 (0.00–7.06)	[14]
		1.40 (0.36–2.43) ^1^	[53]
		1.39 (0.06–2.58) ^1^	[5]
		1.38 (0.35–2.55)	[96]
		0.70 (0.16–1.48) ^1^	[37]
		0.29 (0.05–0.76) ^1^	[95]
Phloretin and phoridzin	Dihydrochalcones	3.13 (1.80–4.46)	[102]
		0.15 (0.01–0.39) ^1^	[95]
		0.09 (0.00–0.33) ^1^	[101]
Luteoin and derivatives	Flavonoids	0.89 (0.00 – 1.80) ^1^	[53]
		0.72 (0.00–1.32) ^1^	[5]

^1^ Values reported on a dry matter (DM) basis in the original studies were converted to a fresh matter (FM) basis assuming 94% moisture content.

**Table 8:** Content of tocopherols (µg/100 g FM) from tomato fruit reported in the literature.

Tomato Fruit Type	α-Tocopherol Mean (Min–Max)	γ-Tocopherol Mean (Min–Max)	Other Tocopherols Mean (Min–Max)	Reference
Salad tomatoes	275 (123–612)	170 (109–306)	α-tocQ: 174 (91–391) β-toc: 43 (trace–117)	[107]
Processing tomatoes	731 (411–1164)	236 (92–451)	α-tocQ: 360 (51–707) β-toc: 50 (26–71)	[107]
Industrial tomato (*Solanum lycopersicon* L., hybrid UG812 J)	1443 (1214–1765) ^1^	13 (0–21) ^1^	α-tocHQ: 445 (223–570) ^1^ α-tocES: 1638(1307–2107) ^1^	[108]
Industrial tomato (UG812 J hybrid)	1158 (761–1471)	NA	NA	[110]
Twenty commercially available ripe tomatoes	2121 (1115–3960)	240 (28–783)	β-toc: 329 (21–1016) δ-toc: 25 (13–38)	[109]

^1^ Values reported on a dry matter (DM) basis in the original studies were converted to a fresh matter (FM) basis assuming 94% moisture content. α-tocQ—α-tocopherylquinone; α-tocHQ—α-tocopheryl hydroquinone; α-tocES—α-tocopheryl succinate; β-toc—β-tocopherol; δ-toc—δ-tocopherol. NA—not available.

**Table 9:** Changes of content of glycoalkaloids (mg/100 g FM) from tomato fruit during ripening [112,113,114,115].

Fruit Maturity Stage	α-Tomatine	Dehydrotomatine	Esculeoside A
Immature green	31–50	8	0–0.5
Mature green	6–30	0.9–4.8	0.5–2
Ripening (breaker–turning)	1–10	0–1	2–12
Ripe red	<0.5 or not detected	not detected	15–25

## Data Availability

No new data were created in this study. All data analyzed in this review are available in the cited publications.
